# Effects of Melt Hydrogenation on the Microstructure Evolution and Hot Deformation Behavior of TiBw/Ti-6Al-4V Composites

**DOI:** 10.3390/ma16062496

**Published:** 2023-03-21

**Authors:** Hui Yan, Liang Wang, Xiaoming Wang, Botao Jiang, Hongcan Liu, Binbin Wang, Liangshun Luo, Yanqing Su, Jingjie Guo, Hengzhi Fu

**Affiliations:** 1 National Key Laboratory for Precision Hot Processing of Metals, School of Materials Science and Engineering, Harbin Institute of Technology, Harbin 150001, China; 2 National Key Laboratory for Remanufacturing, Army Academy of Armored Forces, Beijing 100072, China; uwangxm@126.com

**Keywords:** melt hydrogenation technology, titanium matrix composites, TiB whiskers, microstructure, hot deformation behavior

## Abstract

In this study, Ti-6Al-4V matrix composites reinforced with TiB ceramic whiskers were in situ synthesized and hydrogenated using the melt hydrogenation technique (MHT). The effects of MHT on the microstructure evolution and hot compression behavior of the composites were investigated by optical microscopy (OM), electron backscatter diffraction (EBSD), and transmission electron microscopy (TEM). Hot compression tests were performed at strain rates of 0.1/s, 0.01/s, and 0.001/s and temperatures of 800 °C, 850 °C, and 900 °C; the hot workability of composites significantly improved after hydrogenation, for example, the 900 °C peak flow stress of hydrogenated composites (43 MPa) decreased by 53.76% compared with that of unhydrogenated ones (93 MPa) at a strain rate of 0.01/s. Microstructural observations show that MHT can effectively facilitate the dispersion of TiB whiskers and induce the α/β lath refinement of the matrix in our as-cast hydrogenated composite. During hot compression, MHT effectively promoted the as-cast composite microstructure refinement, accelerated the dynamic recrystallization (DRX) generation, and reduced the stress concentration at the interface between the reinforcement and matrix; in turn, the hydrogenated composites presented low peak stress during hot compression.

## 1. Introduction

Titanium matrix composites (TMCs) are considered promising materials in aerospace, military, navigation, and other fields for their excellent properties such as high strength, low density, and high corrosion resistance; moreover, they have better wear resistance and high-temperature performance than titanium counterparts [1,2,3,4,5,6,7,8]. Without the addition of any alloying elements, the crystal structure of titanium is a hexagonal dense structure (HCP) at room temperature, defined as α-Ti. With the increase in temperature, the allotrope transformation of α-Ti → β-Ti (body-centered cubic structure, BCC) will occur at 882 °C [9]. The addition of alloying elements such as V stabilizes β-Ti at room temperature [1]. In general, duplex-phase titanium alloys with (α + β) have excellent comprehensive properties. Ti-6Al-4V is a widely used (α + β) titanium alloy and has been treated as an excellent matrix material for TMCs [10]. As a ceramic whisker, TiB not only has great thermal stability and high modulus, but also a similar coefficient of thermal expansion to titanium. Furthermore, the in situ synthesized TiB whiskers have a stable and clean bonding interface with the titanium matrix [11,12]. Therefore, interest in in situ TiBw-reinforced Ti-6Al-4V composites has been growing over the past few years. Several processing techniques for the in situ synthesis of TiBw-reinforced titanium matrix composites have been developed, including powder metallurgy (PM) [13], spark plasma sintering (SPS) [14], self-propagating high-temperature synthesis (SHS) [15], selective laser melting (SLM) [16], and ingot metallurgy (IM) [17]. Due to the advantages of microstructure control and near net shape machining, these powder-based manufacturing technologies can produce customized products in industry sectors, but the expensive powder costs and lengthy process flow cannot meet the production demand of large size, heavy weight, and large-scale parts [18]. The main advantages of traditional ingot metallurgy processing are as follows: the technical scheme is mature, the production cost is low, and the process flow is short. However, the as-cast ingots always have many defects and low mechanical properties due to the inhomogeneous microstructure. To eliminate casting defects, refine grains, and improve mechanical properties, TMCs ingots usually need multiple steps of thermo-treatment and solid deformation [19,20]. In addition, the reinforcements could be easily broken in these post-processes, and the interface between hard ceramic particles and soft matrix alloy will be prone to crack due to the stress concentration.

Thermohydrogen treatment (THT), which uses hydrogen as a temporary alloying element, can greatly improve the hot workability and refine the microstructure of titanium-based alloys [21,22]. In this process, hydrogen diffuses into the titanium alloy by holding the material at a relatively high temperature under a hydrogen-containing atmosphere for several hours. Finally, the hydrogen is removed by a simple vacuum (or inert gas) anneal [23,24]. Niu et al. [25] found that the superplastic deformation domain occurred at a lower deformation temperature and higher strain rate of hydrogenated Ti600 alloy, spread over a wider temperature range than that of unhydrogenated Ti600 alloy. Ma et al. [26] concluded that hydrogen can significantly decrease the flow stress of TiAl alloys, which was decreased by 40% after hydrogenation with 0.043 wt.% H under a strain rate of 0.01 s^−1^ at 1200 °C. Lu et al. [27] discovered that hydrogen significantly decreased the flow stresses and the deformation temperature of (TiB + TiC)/Ti-6Al-4V composites after THT, enabling the composites to deform at a higher strain rate at the same flow stress level. Nevertheless, hydrogen concentrations exhibit heterogeneity in large samples or engineering components after THT, and the high cost also limits its applications [28]. To overcome these shortcomings, we added hydrogen to the metallurgical process, which involved smelting raw materials under an atmosphere of hydrogen/argon mixture, termed Melt Hydrogenation Technology (MHT) [29]. MHT can simultaneously complete the synthesis and hydrogenation of titanium-based materials, including titanium alloys [30,31], γ-TiAl alloys [32,33], and TMCs [34,35,36,37]. In the literature [34], the hot workability of (TiB + TiC)/Ti-6Al-4V composite prepared by MHT was investigated; it was found that hydrogen induced the competing of softening and hardening in the hydrogenated TMCs at different deformation temperatures and that MHT improved the motion coordination between the reinforcements and matrix during hot compression. The literature [36] discussed the improved hot workability of TiC/Ti-6Al-4V composite prepared by MHT and found that hydrogen significantly increased the size of eutectic TiC particles and prior β grains, reduced the titanium atom bonding force, decreased the β/(α + β) transition temperature, increased the ratio of DRX, and improved the dislocation mobility. However, these studies focus more on the effects of MHT on the reinforcement or matrix and the changes produced in the matrix during hot compression, but do not design and explore the role played by their interface during hot compression deformation. The literature [37] investigated the atomic arrangement at the interface and the orientation of TiB and α-Ti in hydrogenated TiB/Ti6Al4V composites. It is believed that the addition of hydrogen accelerated atomic diffusion during solidification, so in the hydrogenated TMC, the transition layer of interface became thinner, the atoms were arranged more orderly, the interface bonding strength was improved, and the crystal defects were eliminated. However, the deformation properties’ improvement mechanism of hydrogenated TMC has not been comprehensively summarized by analyzing the strain on both sides of the interface.

There are still few relevant studies on the effects of MHT on the microstructure and mechanical properties of TMCs, and a systematic study on the improvement of the hot workability of TiBw/Ti-6Al-4V by MHT has not been established. Based on the previous background, in this paper, 2.5 vol.% TiB whisker (TiBw)-reinforced Ti-6Al-4V matrix composites were prepared under a H_2_/Ar mixed atmosphere. The unique effects of MHT on the reinforcement, matrix, and the interface between the reinforcement and matrix were analyzed. The hot compression behavior and as-compressed microstructure evolution of the composites with different hydrogen contents were also investigated at different deformation temperatures and strain rates. The present study allows a comprehensive investigation of the mechanism of MHT refinement of TMC microstructure and improvement of hot workability.

## 2. Materials and Methods

### 2.1. Preparation

The raw materials were commercial titanium sponge (99.9% purity, Fushun Titanium Industry Ltd., Fushun, China), high-purity aluminum particles (99.99% purity, ZhongShi Advanced Materials Ltd., Beijing, China), Al-V alloy particles (99.9% purity, 58.18 wt.% vanadium, ZhongShi Advanced Materials Ltd., Beijing, China), and TiB_2_ powder (99.9% purity, Beijing Ryubon New Material Technology Ltd., Beijing, China). TiBw/Ti-6Al-4V matrix composite ingots were synthesized in a high-vacuum plasma arc melting furnace equipped with a non-consumable tungsten electrode and a water-cooled copper crucible (Shenyang Hotstar New Materials Preparation Technology Ltd., Shenyang, China). The process of preparing hydrogenated TMC was as follows: The materials were stacked in the crucible in order from low to high melting point; then, after evacuating to 5 × 10^−3^ Pa, a gas mixture with a volume ratio of Ar:H_2_ = 3:2 was inflated into the furnace and the melting began. The ratio of hydrogen was controlled by a JF-2200 system, which had the real-time display of hydrogen partial pressure. The process of preparing unhydrogenated TMC was the same as above, with only argon in the gas inflation process. All the ingots were remelted four times to maintain chemical uniformity. Each specimen for microscope observation or hot compression testing was cut from the center of the ingot. The hydrogen contents of TMCs were measured by an oxygen–hydrogen analyzer (ROH600, LECO, St. Joseph, MI, USA). The chemical composition of each material is shown in Table 1.

### 2.2. Characterization

An X-ray diffractometer (XRD) (Empyrean, Panalytical, Almelo, The Netherlands) was used to identify the phases of the composites. An optical microscope (OM) (GX53, OLYMPUS, Tokyo, Japan) was used for the surface topography observation of the composites. The specimens for surface topography observation were mechanically ground, polished, and then etched in a solution (volume ratio of HF:HNO_3_:H_2_O = 1:1:8) for 20 s. An electron backscatter diffraction (EBSD) analysis of the composites was conducted on a scanning electron microscope (SEM) (SUPRA55, ZEISS, Jena, Germany), and the data were processed by AztecCrystal software (version 2.1, Oxford, UK). The specimens for EBSD were electrochemically polished in an electrolyte solution (volume ratio of HClO_4_:CH_3_(CH_2_)_3_OH:CH_3_OH = 6:34:60) to reduce the surface stress. A transmission electron microscope (TEM) (Talos F200X, FEI, Hawthorne, CA, USA) was used to observe the microstructure of the as-compressed composites. The specimens were first mechanically ground to 50 µm and then ion milled (PIPS II 695, GATAN, Pleasanton, CA, USA) for TEM observation.

### 2.3. Testing

The temperature of the β/(α + β) phase transition of as-cast TMCs was tested by differential scanning calorimetry (DSC) (STA 449, NETZSCH, Selb, Germany). The specimen size used for the DSC test was 3 mm in diameter and 3.5 mm in height. The surface of the specimens was polished to remove the oxide layer before the test. The temperature range was from 25 to 1200 °C with a heating and cooling rate of 20 °C/min. The phase transition temperature was 941 °C and 918 °C for the unhydrogenated and hydrogenated composites, respectively. Accordingly, the hot compression temperatures (800 °C, 850 °C, and 900 °C) used in this study are in the (α + β) phase region.

The specimen was processed into a cylinder with a diameter of 6 mm and a height of 9 mm for a hot compression test. The hot compression tests were performed on a dynamic thermodynamic simulator (AGXplus, SHIMADZU, Kyoto, Japan) at a strain rate of 0.1/s, 0.01/s, and 0.001/s, with temperatures of 800 °C, 850 °C, and 900 °C. The specimens were heated to the setting temperature at 10 °C/s and then held for 3 min. Before hot compression, the ends and sides of the specimen were sandpapered to remove the oxide layer. Graphite gaskets were placed between the ends of the specimen and the indenter to ensure lubricity. To prevent hydrogen escaping from the specimen during the test, the specimen was protected with silica gel. The deformation temperature was measured by thermocouples. After hot compression, the specimens were water-quenched immediately to retain the microstructure.

The hardness values of the as-cast and as-compressed specimens were determined by a Vickers’s hardness testing apparatus (HVS-1000A, Huayin, China) with an operating load of 1.96 N and a dwell time of 15 s. Since the hardness values of the reinforcement and the matrix are very different, the sample was measured at least 5 times and the average value was reported.

## 3. Results and Discussion

### 3.1. As-Cast Microstructure Characteristics

**Figure 1:**
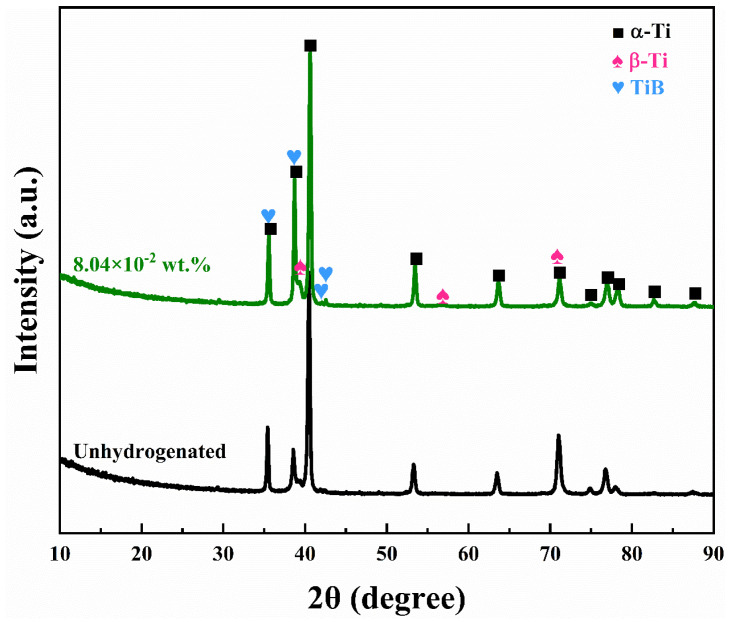
XRD patterns of TMCs with different hydrogen contents.

Figure 1 shows the XRD patterns of the TiBw/Ti-6Al-4V composite with different hydrogen contents. TMCs were fabricated based on the chemical reactions: Ti + TiB_2_ → TiB. All the TMCs contained only α-Ti, β-Ti and TiB. No diffraction peaks of TiB_2_ or TiH_x_ were found, indicating that TiB_2_ was completely reacted with Ti by MHT. TMCs with different hydrogen contents were successfully prepared.

**Figure 2:**
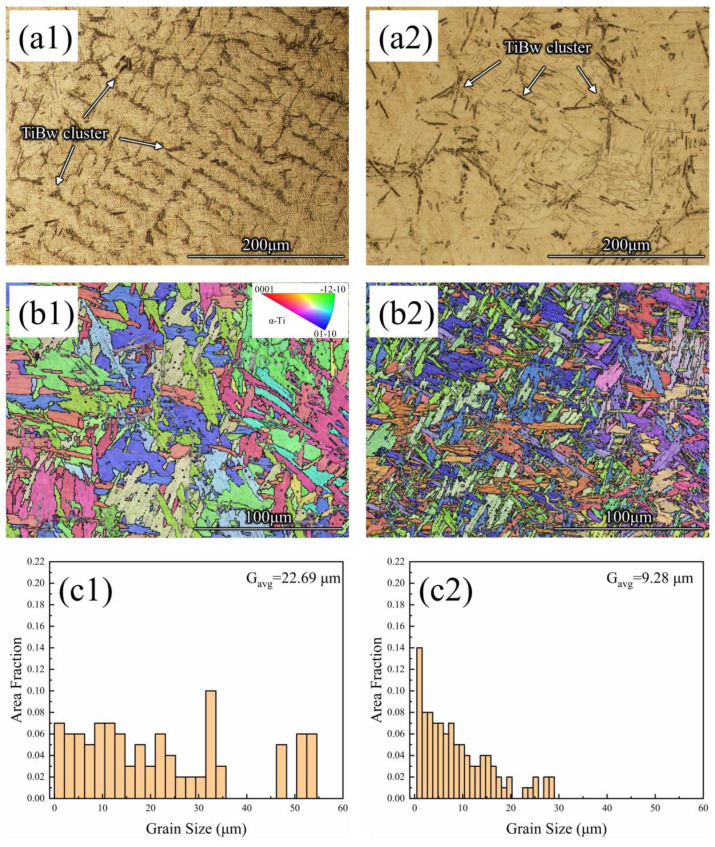
Microstructure of as-cast TMC: (**a1**,**a2**) images of OM, (**b1**,**b2**) IPF maps of EBSD, (**c1**,**c2**) histogram of grain size, (**a1**,**b1**,**c1**) unhydrogenated TMC, (**a2**,**b2**,**c2**) hydrogenated TMC.

The OM images and inverse pole figure (IPF) maps in Figure 2 show the distribution of reinforcements as well as the surface topography of as-cast TMCs. After the TMCs were etched, the ceramic reinforcements protruded slightly on the surface and showed a dark contrast, as shown in Figure 2(a1,a2). The TiBws embedded in the matrix alloy were similar to slender needles because TiB crystal grows fastest in the [010] direction [38]. According to the Ti-B binary phase diagram [08], the B content in this study belongs to the sub-eutectic composition, so that the TiBws formed in this study were eutectic. Specifically, during solidification, β-Ti grains first nucleated and grew into dendrites, the liquid phase was consumed, and the eutectic reaction (L → Ti + TiB) occurred between the β-Ti dendritic arms. Therefore, these clustered eutectic TiBws displayed the prior β-Ti grain shape. Finally, when the temperature dropped to the β-Ti→α-Ti phase transition point, most of the prior β-Ti transformed to α-Ti, and these α-Ti lamellae and residual β-Ti formed α/β lath, as shown in Figure 2(b1,b2). Generally, α” phase (orthorhombic) is very common in some (α + β) titanium matrix composite, which is obtained by the rapid cooling of β phase, such as quenching [39]. Others are stress-induced β to α” phase transformation [40]. The as-cast TMCs used in this study were prepared by melting in a water-cooled copper crucible and then cooling in a furnace. Although the water-cooled copper crucible has a fairly good cooling effect, the solidification rate is ~10^2^ K/s. In comparison with the literature [41], the TiB/Ti-6Al-4V composite prepared by SLM also showed no α” phase, while the solidification rate value was as high as ~10^6^–10^8^ K/s. Therefore, it can be assumed that the composites prepared in this study had almost no α” phase.

Comparing Figure 2(a1,a2), it can be found that the unhydrogenated titanium alloy matrix was composed of long dendrites and the TiBws were tightly clustered, while in hydrogenated TMC, the matrix consisted of more equiaxed dendrites and had a lower degree of aggregation of TiBws and more interstices between them. In other words, MHT can effectively reduce the degree of aggregation during the solidification of TiBws and uniformly distribute their reinforcement. The temperature of the Ar-H2 plasma arc was hundreds of degrees higher than that of the Ar plasma arc only; that is, hydrogen caused more overheating at the molten composite surface [42,43,44]. Due to the same heat dissipation conditions of the water-cooled copper crucible, there was a larger temperature gradient in the hydrogenation molten pool. In the hydrogenation molten pool with a higher temperature gradient, as the crystals nucleated and grew into the higher temperature melt, some of the longer dendrite arms partially melted and fell off, becoming nucleated sites again in the molten pool. More nucleation sites constrained the growth of the crystals, so smaller, more equiaxed dendrites appeared in the hydrogenated TMC.

Figure 2(c1,c2) shows the histogram of grain size in Figure 2(b1,b2). It can be seen that the average grain size of the hydrogenated TMC was smaller than that of the unhydrogenated TMC, with a grain size of 22.69 μm for the unhydrogenated composite and 9.28 μm for the hydrogenated TMC. On the one hand, as previously analyzed, this is due to the formation of smaller sized equiaxed β-Ti dendrites in the hydrogenated TMC during solidification, and smaller α/β lath formed during the subsequent phase transformation. On the other hand, the more diffuse TiBws acted as nucleation sites for the β-Ti → α-Ti phase transition, which improved the nucleation of α-Ti and constrained the growth of α-Ti. Therefore, MHT can effectively improve the microstructure of as-cast TMC, not only refining the grain of the matrix alloy, but also reducing the aggregation of the reinforcement. Further, the Vickers hardness of as-cast unhydrogenated TMC and hydrogenated TMC was measured, and the values were 194 HV and 246 HV, respectively. In other words, MHT can not only effectively refine and homogenize the microstructure of TMCs, but also improve its mechanical properties, therefore improving the quality of ingot metallurgy.

### 3.2. Hot Compression Behavior

**Figure 3:**
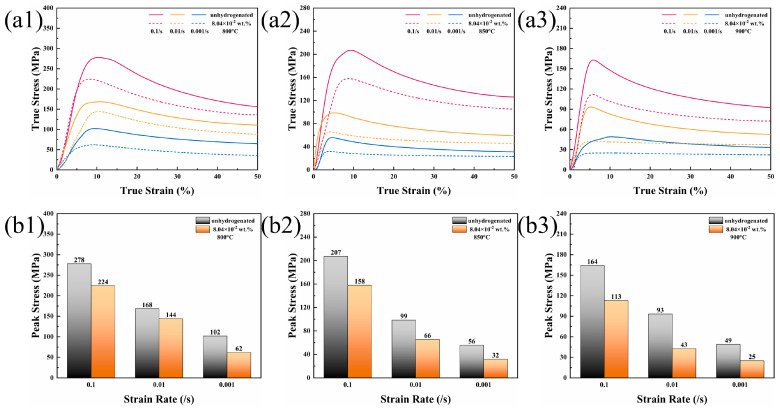
True stress−strain curves and peak stress of TMC: (**a1**,**b1**) 800 °C, (**a2**,**b2**) 850 °C, (**a3**,**b3**) 900 °C.

Figure 3(a1,a2,a3) plots the true stress–strain curve of TMC under temperatures of 800 °C, 850 °C, and 900 °C, and three strain rates. The parameters are represented in the upper right corner of each chart. These curves show typical dynamic recrystallization (DRX) characteristics. In the early stage of hot compression, dislocation density increased sharply, and DRX did not or rarely occurred due to small strain. Work hardening played a dominant role in this period, and the stress also increased sharply. After the peak stress was reached, the composite softened due to the acceleration of DRX, showing a mild decrease in stress. Figure 3(b1,b2,b3) summarizes the peak stress of unhydrogenated and hydrogenated TMC under corresponding hot compression conditions. It can be found that the peak stress of all the composites decreased with the increase in temperature and the decrease in strain rate. For instance, at the rate of 0.1/s and under temperatures of 800 °C, 850 °C, and 900 °C, the peak stresses of unhydrogenated TMC were 278 MPa, 207 MPa, and 164 MPa, and those of hydrogenated TMC were 224 MPa, 158 MPa, and 113 MPa, respectively. Similarly, when the temperature was 900 °C and the strain rate was 0.1/s, 0.01/s and 0.001/s, the peak stresses of unhydrogenated TMC were 164 MPa, 93 MPa, and 49 MPa, while those of hydrogenated TMC were 113 MPa, 43 MPa, and 25 MPa, respectively. This is consistent with the results in the literature [27], indicating that the decrease in the peak stress of TMC with increasing temperature and decreasing strain rate is an intrinsic property of the material. Under each hot compression condition, the hydrogenated TMC showed lower peak stress. At a temperature of 900 °C and a strain rate of 0.01/s, the hydrogen-induced peak stress decreased most significantly; the peak flow stress of hydrogenated composite (43 MPa) decreased by 53.76% compared with that of unhydrogenated composite (93 MPa), indicating that MHT can effectively reduce the peak stress during hot compression and improve the hot workability of in situ TiBw-reinforced Ti-6Al-4V composites.

### 3.3. As-Compressed Microstructure Observation

**Figure 4:**
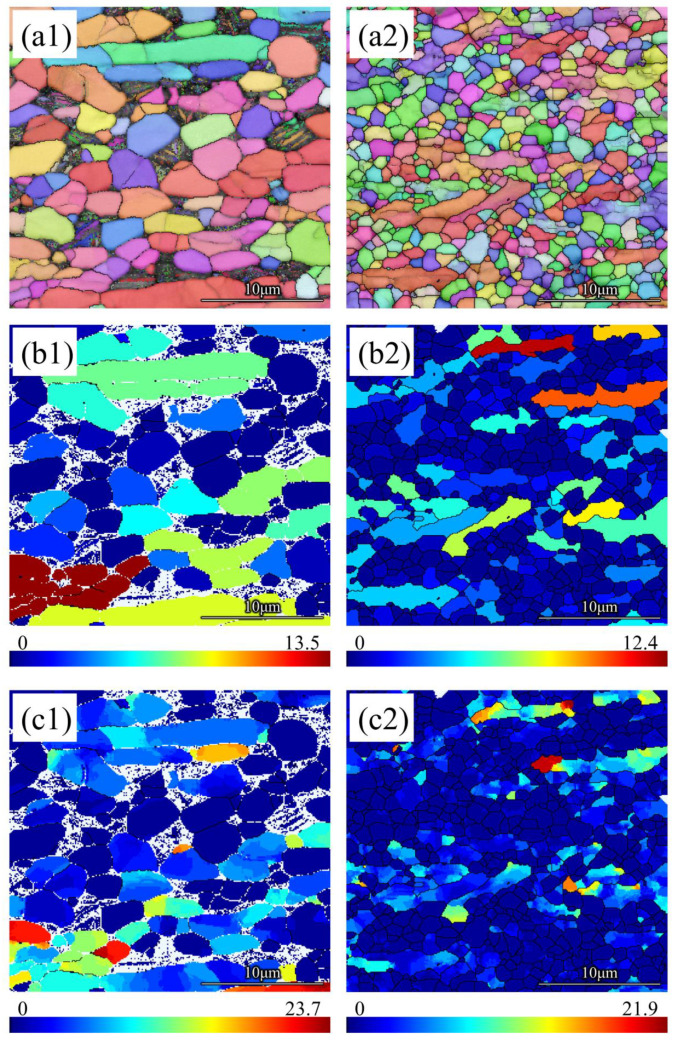
Microstructure of as-compressed TMC: (**a1**,**a2**) IPF maps, (**b1**,**b2**) GAM maps, (**c1**,**c2**) GROD maps, (**a1**,**b1**,**c1**) unhydrogenated TMC, (**a2**,**b2**,**c2**) hydrogenated TMC.

The microstructures of TMCs compressed at a temperature of 800 °C and at the rate of 0.01/s, analyzed by EBSD, are shown in Figure 4, where (a1,a2) are IPF maps; (b1,b2) are grain average misorientation (GAM) maps, which show the average value of each grain orientation dispersion; and (c1,c2) are grain reference orientation deviation (GROD) maps, which show the deviation between the orientation of each pixel point inside a grain and the average orientation of this grain.

Figure 4(a1,a2) shows that after hot compression, the average grain size was 4.5 μm for unhydrogenated TMC and 2.1 μm for hydrogenated TMC. Equiaxed grains and elongated grains were observed in both unhydrogenated and hydrogenated TMCs. Combined with Figure 4(b1,b2,c1,c2), it can be found that the GAM and GROD of equiaxed grains were smaller than that of elongated grains, which indicates that the equiaxed grains were DRX grains, while the elongated grains were deformed grains. Comparing the percentage of equiaxed grains in unhydrogenated and hydrogenated TMC, it can be found that more DRX grains appeared in hydrogenated TMC. Since there were almost no dislocations in the DRX grains, the dislocation slip had a lower resistance, which softened the composite and reduced the flow stress. Therefore, the hot compression peak stress of hydrogenated TMC was lower.

During the hot compression process, a large number of dislocations accumulate in the initial stage, and when the dislocation density is high enough, the DRX process occurs under the effect of thermal activation. DRX eliminates deformation defects such as dislocations and sub-grain boundaries in deformation by the nucleation and growth of DRX grains, and this process is realized by the migration of high-angle grain boundaries [34]. The small grain size of the hydrogenated composite matrix can accumulate a large number of dislocations in the initial stage of hot compression, and boundaries of fine grains also promote the nucleation of DRX grains. The generation of a large number of DRX grains eliminated the dislocations and decreased the flow stress of hydrogenated composite, so the hydrogenated composite had a lower peak stress. In the subsequent process, these large numbers of DRX grains constrained each other, and the DRX grain size of the final hydrogenated TMC was also smaller than that of the unhydrogenated TMC. The Vickers hardness value for the unhydrogenated TMC and hydrogenated TMC after hot compression was 296 HV and 355 HV, respectively. This implies that the Vickers hardness increased with decreasing grain size and was consistent with the results of the DRX grains in the IPF map.

**Figure 5:**
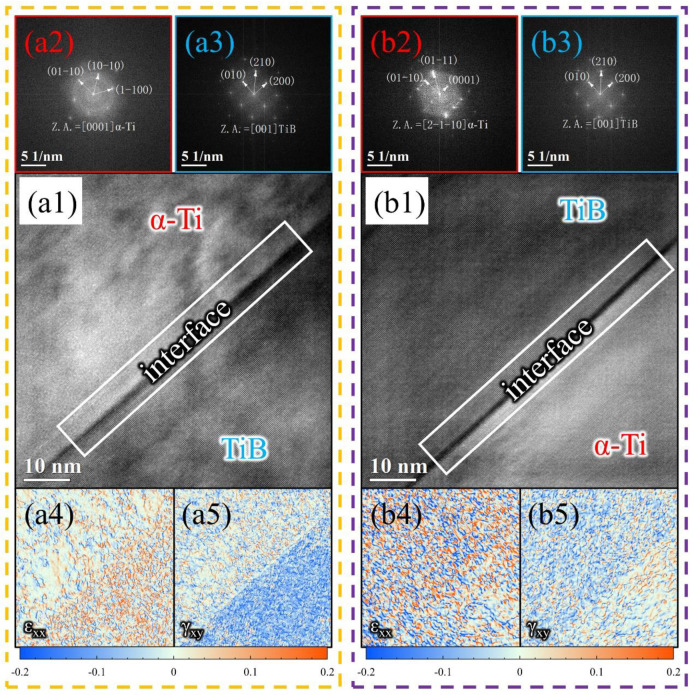
Structure of the interface in the as-compressed TMCs: (**a1**,**b1**) high-resolution images, (**a2**,**a3**,**b2**,**b3**) SAED patterns, (**a4**,**a5**,**b4**,**b5**) GPA maps, (**a1**–**a5**) unhydrogenated TMC, (**b1**–**b5**) hydrogenated TMC.

Figure 5 shows the structure of the interface between the reinforcement and the matrix alloy in the as-compressed TMCs, where (a1,b1) are high-resolution images; (a2,a3) and (b2,b3) are selected area electron diffraction (SAED) patterns; (a4,a5) and (b4,b5) are geometric phase analysis (GPA) maps, which show the strain distribution of ε_xx_ and γ_xy_ at the interface; and the positive value is for compressive strain and the negative for tensile strain.

It can be found that the unhydrogenated and hydrogenated TMCs had a greater difference in the interfacial strain state after hot compression. From the comparison of ε_xx_ and γ_xy_, it can be seen that the difference in the degree of lattice deformation between α-Ti and TiB in the unhydrogenated TMC was larger, and TiB had a higher degree of strain with a larger strain gradient at the interface. The difference in the degree of lattice deformation between α-Ti and TiB in hydrogenated TMC was smaller, as was the strain gradient at the interface. The presence of the hard ceramic reinforcements impeded the flow of the matrix alloy during hot compression and generated a large amount of dislocation accumulation at the reinforcement interface, leading to stress concentration at the interface. Further, the analysis of GPA indicates that MHT can effectively reduce this stress concentration that may lead to deformation defects, thus improving the hot workability of the composites.

GPA can well analyze the accumulation of dislocations at the interface between the reinforcement and matrix, so as to infer the stress concentration during hot compression deformation. However, GPA is a static analysis technology with a small scope, and the calculated results are semi-quantitative, which is limited to the current state of the art. Therefore, it can help researchers understand the hot deformation optimization mechanism of hydrogenated TMC theoretically, but it is difficult to become a practical application in engineering. Therefore, the mechanism of MHT refining the microstructure and improving the hot workability of TMC still has great room for exploration.

## 4. Conclusions

We used MHT to in situ synthesize and hydrogenate 2.5 vol.% TiBw-reinforced Ti-6Al-4V matrix composites. We investigated the effect of MHT on the microstructure evolution and hot deformation behavior of TMCs. Based on the experimental results, we can draw the following conclusions:

(1) MHT can homogenize the distribution of reinforcements, reduce the grain size of the matrix alloy, and refine the as-cast microstructure of TMC.
(2) MHT can effectively reduce the peak stress during hot compression. At a temperature of 900 °C and a strain rate of 0.01/s, the hydrogen-induced peak stress decreased most significantly, with a reduction of 53.76%.
(3) MHT refinement of the as-cast microstructure can greatly promote DRX grain nucleation, inhibit DRX grain growth, reduce stress concentration at the interface, and thus, improve the hot workability of TMC.

## Figures and Tables

**Table 1:** Chemical composition for TMCs.

Element (wt. %)	Al	V	B	H	Ti
Unhydrogenated TMC	5.91	3.94	0.48	0	Balanced
Hydrogenated TMC	5.90	3.93	0.48	0.0804	Balanced

## Data Availability

Some or all data, models, or code that support the findings of this study are available from the corresponding author on reasonable request.
